# Serine Protease PRSS23 Is Upregulated by Estrogen Receptor α and Associated with Proliferation of Breast Cancer Cells

**DOI:** 10.1371/journal.pone.0030397

**Published:** 2012-01-23

**Authors:** Hau-Shien Chan, Shing-Jyh Chang, Tao-Yeuan Wang, Hung-Ju Ko, Yu-Chih Lin, Kuan-Ting Lin, Kuo-Ming Chang, Yung-Jen Chuang

**Affiliations:** 1 Department of Medical Science, Institute of Bioinformatics and Structural Biology, National Tsing Hua University, Hsinchu, Taiwan, R.O.C.; 2 Institute of Molecular and Cellular Biology, National Tsing Hua University, Hsinchu, Taiwan, R.O.C.; 3 Department of Obstetrics and Gynecology, Hsinchu Mackay Memorial Hospital, Hsinchu, Taiwan, R.O.C.; 4 Department of Pathology, Mackay Memorial Hospital, Taipei, Taiwan, R.O.C.; 5 Mackay Medical College, Taipei, Taiwan, R.O.C.; 6 Mackay Medicine, Nursing and Management College, Taipei, Taiwan, R.O.C.; 7 Department of Life Science, National Tsing Hua University, Hsinchu, Taiwan, R.O.C.; 8 Department of Pathology, Hsinchu Mackay Memorial Hospital, Hsinchu, Taiwan, R.O.C.; Baylor College of Medicine, United States of America

## Abstract

Serine protease PRSS23 is a newly discovered protein that has been associated with tumor progression in various types of cancers. Interestingly, *PRSS23* is coexpressed with estrogen receptor α (ERα), which is a prominent biomarker and therapeutic target for human breast cancer. Estrogen signaling through ERα is also known to affect cell proliferation, apoptosis, and survival, which promotes tumorigenesis by regulating the production of numerous downstream effector proteins.

In the present study, we aimed to clarify the correlation between and functional implication of ERα and PRSS23 in breast cancer. Analysis of published breast cancer microarray datasets revealed that the gene expression correlation between ERα and PRSS23 is highly significant among all ERα-associated proteases in breast cancer. We then assessed PRSS23 expression in 56 primary breast cancer biopsies and 8 cancer cell lines. The results further confirmed the coexpression of PRSS23 and ERα and provided clinicopathological significance. *In vitro* assays in MCF-7 breast cancer cells demonstrated that PRSS23 expression is induced by 17β-estradiol-activated ERα through an interaction with an upstream promoter region of *PRSS23* gene. In addition, PRSS23 knockdown may suppress estrogen-driven cell proliferation of MCF-7 cells.

Our findings imply that PRSS23 might be a critical component of estrogen-mediated cell proliferation of ERα-positive breast cancer cells. In conclusion, the present study highlights the potential for PRSS23 to be a novel therapeutic target in breast cancer research.

## Introduction

Bioinformatics approaches have shown that the serine protease 23 gene (*PRSS23*) is highly conserved in vertebrates and is predicted to encode a novel protease on chromosome 11q14.1 in humans [1], [2], [3]. Previous expression-profiling studies have suggested that enhanced PRSS23 expression is observed in various types of cancers, including breast [4], [5], [6], prostate [7], papillary thyroid [8], and pancreatic cancers [9], and the expression of the PRSS23 has been linked with tumor progression in human [1]. In addition, studies in MCF-7/BUS cells revealed that mRNA level of PRSS23 may be stimulated by estrogen and reduced by tamoxifen treatment [5], [10].

Estrogen, which are well conserved in vertebrates, represents a group of sex steroid hormones that include estradiol, estrone, and estriol [11]. Although estrogen is the predominant sex hormone in females, its levels are relatively low in males. Along with its role in reproduction, estrogen also affects many cellular functions during development and in adulthood. Ample evidence has shown that estrogen and anti-estrogen agents, such as tamoxifen and fulvestrant, can specifically bind to the ligand binding domain of estrogen receptor α (ERα) to modulate differential expression of downstream transcriptional targets of ERα in breast cancer cells. These findings suggest that ERα could be a vital prognostic biomarker in breast cancer [12], [13], [14], [15], [16], [17].

Collective evidence suggests that estrogen signaling regulate a variety of biological processes [18]. For instance, estrogen signaling plays a pivotal role in growth and development of mammary glands which is consistent with its role in normal sexual and reproductive functions. Indeed, canonical estrogen signaling affects the expression of specific downstream effector genes that enhance cell survival via anti-apoptotic pathways. In addition, estrogen signaling increases proliferation of breast cancer cells by upregulating expression of cell cycle enhancers (e.g., cyclin D1) and transcription factors (e.g., c-myc and E2F) expression in breast cancer [19], [20]. Although importance of novel ERα-related proteases to breast cancer progression is unclear, we hypothesized that estrogen could also enhance breast cancer cell progression through intracellular proteases.

In the present study, we investigated the gene expression of the ERα-related proteases in breast cancers. Our results indicate that there was a high level of PRSS23 expression in ERα-positive breast cancer cells. In addition, *in vitro* assays revealed that PRSS23 expression was upregulated at the transcriptional level by ERα and was associated with breast cancer cell proliferation. Thus, PRSS23 might be a novel target for adjuvant therapy for breast cancer progression.

## Results

### PRSS23 mRNA levels are correlated with ESR1 mRNA expression in breast cancer

**Figure 1:**
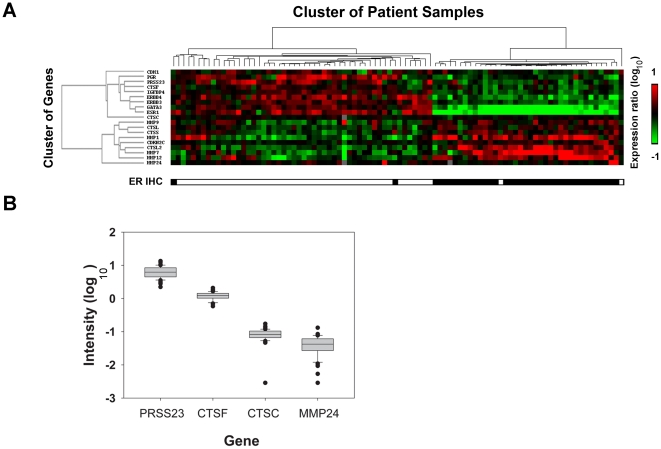
Gene expression analysis of breast cancer patients. **A.** Clustering of self-organizing maps was done to analyze gene expression of proteases, ESR1 and ESR1-coregulated genes among 90 breast cancer patients. The red-colored boxes represent upregulated genes (ratio of log_10_ intensity), and the green-colored boxes indicate downregulated genes. The cluster to the left shows the hierarchy relationship of gene expression patterns, and the cluster at the top indicates correlation among groups of patient samples. The lowest box represents corresponding immunohistochemistry results of ERα staining for each sample (open is positive, and filled is negative). **B.** The box plot showed expression intensity of PRSS23, CTSF, CTSC, and MMP24 in 52 ERα-positive breast cancer specimens.

Our first aim was to screen for novel proteases that are coregulated with ERα in breast cancer by mining the microarray dataset published by van't Veer et al. [21] Proteases including CTSC (cathepsin C), CTSF, CTSL, CTSS, CTSL2, MMP-1 (matrix metalloprotease-1), MMP-7, MMP-9, MMP-12, MMP-24, and PRSS23 that were associated with ESR1(mRNA of ERα) expression. We then used hierarchy of correlation clustering to examine the correlations between ESR1 and the candidate protease genes. As shown in Fig. 1A, self-organized map analysis revealed that the gene expression profiles of PRSS23, CTSC, and CTSF were clustered within the group of ESR1 coregulated genes. Other well-known estrogen-upregulated genes, like CDH (E-cadherin), PGR (progesterone receptor), ERBB3 (V-erb-b2 erythroblastic leukemia viral oncogene homolog 3), ERBB4, and GATA3 (GATA binding protein 3), were also found in the same cluster. By comparison, CDKN2C (cyclin-dependent kinase inhibitor 2C, p18), MMP-1, MMP-7, MMP-9, MMP-12, MMP-24, CTSL, CTSL2, and CTSS were negatively correlated with ESR1 mRNA levels. These findings were consistent with those from regression analyses by van't Veer et al.

We also compared the expression intensities of PRSS23, CTSC, CTSF, and MMP-24 from 52 ERα-positive breast cancer specimens within the van't Veer dataset. The average expression levels (log_10_ intensity) of PRSS23, CTSF, CTSC and MMP-24 were 0.779, 0.075, −1.101, and −1.434, respectively (Fig. 1B). In addition to being significantly coregulated with ESR1 expression, the present results suggest that there is greater mRNA expression level of PRSS23 in breast cancer specimen than other well-known cancer-related proteases. Because the expression of PRSS23 in breast cancer has not been clearly characterized, we targeted PRSS23 for further analysis in the present study.

### High PRSS23 expression was observed in ERα-positive breast cancer cells from breast cancer patients

To enable the detection of the PRSS23 protein, we raised an antibody against PRSS23 by injecting recombinant GST-PRSS23 protein into a rabbit. After standard purification (the detailed procedure is described in Materials and Methods S1), we validated the efficacy and specificity of this custom anti-PRSS23 antibody by immunoblot of protein from MCF-7 cells with or without ectopic PRSS23 overexpression. Both endogenous and overexpressed PRSS23 could be detected as a double-band pattern around 47 kDa (Fig. S1), which is close to PRSS23's hypothetical molecular weight (43 kDa).

**Figure 2:**
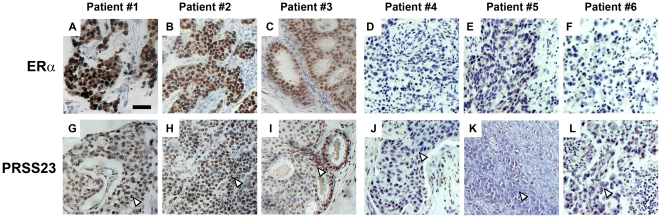
Expression of ERα and PRSS23 in human breast carcinoma. Immunohistochemical analysis revealed expression level of ERα (**A**, **B**, **C**, **D**, **E**, **F**) and the corresponding PRSS23 expression of the same sample (**G**, **H**, **I**, **J**, **K**, **L**) in 6 different breast cancer specimens. The scale bar is 50 µm.

We used the custom anti-PRSS23 antibody to perform immunohistochemical assays on cancer specimens from 56 primary breast tumors collected in Taiwan. Interestingly, PRSS23 expression was detected in the nuclei of malignant breast tumor tissues. To validate the relationship between PRSS23 and ERα expression, we selected 6 representative sets of tumor samples from breast cancer patients that were either ERα-positive (Fig. 2A, B, C) or ERα-negative (Fig. 2D, E, F). Upon close examination, PRSS23 expression was found to be much higher in the nucleoplasm of ERα-positive breast cancer specimens (Fig. 2G, H, I) compared with the nucleoplasm of ERα-negative breast cancer specimens (Fig. 2J, K, L).

For systemic comparison, the staining intensity of anti-PRSS23 in 56 Taiwan breast cancer samples was classified as strong (Fig. S2A), moderate (Fig. S2B), or weak (Fig. S2C). This was performed by comparing the staining intensity in the cancer specimens to the intensity in normal cells in the vicinity of tumor tissues. Specifically, we characterized PRSS23 staining by comparing PRSS23 expression intensities in the nucleoplasm of cancer cells to the expression intensities in normal stromal cells and endothelial cells using the Allred immunohistochemistry score system [22]. Based on the assigned total Allred scores, we grouped the 56 breast cancer specimens into two cohorts: high PRSS23 expression (total Allred score>3), and the low PRSS23 expression (total Allred Score 0–3) (Table 1). Strikingly, we found that nearly 75% of the ERα-positive breast cancer samples from Taiwanese patients are belonged to the group with high PRSS23 expression (Allred score>3). Conversely, over 80% of the ERα-negative breast cancer samples belonged to the low PRSS23 expression group (Allred score≤3). Statistical analyses also indicated that increased PRSS23 expression was significantly correlated with ERα status of the cells (n = 56, *p* = 0.005).

**Table 1:** Clinicopathological characteristics and PRSS23 expression profile of breast cancer specimens.

	Low PRSS23 expression (score 0–3†)	Higher PRSS23 expression (score>3†)	*p* ‡
Total	28	28	N.S.
Tumor size (cm)*	2.95±0.40	3.12±0.51	0.79.
Lymph node invasion			
+	10	7	0.56
−	18	21	
ERα (IHC)			
+	6	22	0.0001
−	22	6	
HER2 (IHC)			
+	13	7	0.79.
−	15	21	
Histological type			
Ductal carcinoma	22	25	.
lobular carcinoma	0	0	
Others	6	3	

*Data are mean ± SD.

† Total Allred score of proportion score and intensity score.

‡ *p* assessed using Fisher's exact probability test.

Taken together, the results derived from the clinicopathological and immunohistochemical analyses imply that PRSS23 expression is closely related to ERα expression (Table 1). Interestingly, we did not find any statistical significance between PRSS23 expression and tumor invasion (*p* = 0.56) or PRSS23 and HER-2 overexpression, which suggests that HER-2 amplification may not affect PRSS23 expression (*p* = 0.79).

### PRSS23 is highly expressed in ERα-positive breast cancer cell lines

Subsequently, we measured both mRNA and protein levels of PRSS23 in 8 different human cell lines: 3 ERα-positive breast cancer cell lines (MCF-7, BT-474, T-47D), 2 ERα-negative breast cancer lines (Hs.578t, MDA-MB-231), 1 mammary epithelial cell line (MCF-10A), 1 endometrial cell line (RL95-2), and 1 cervical cancer line (Ca-SKi).

**Figure 3:**
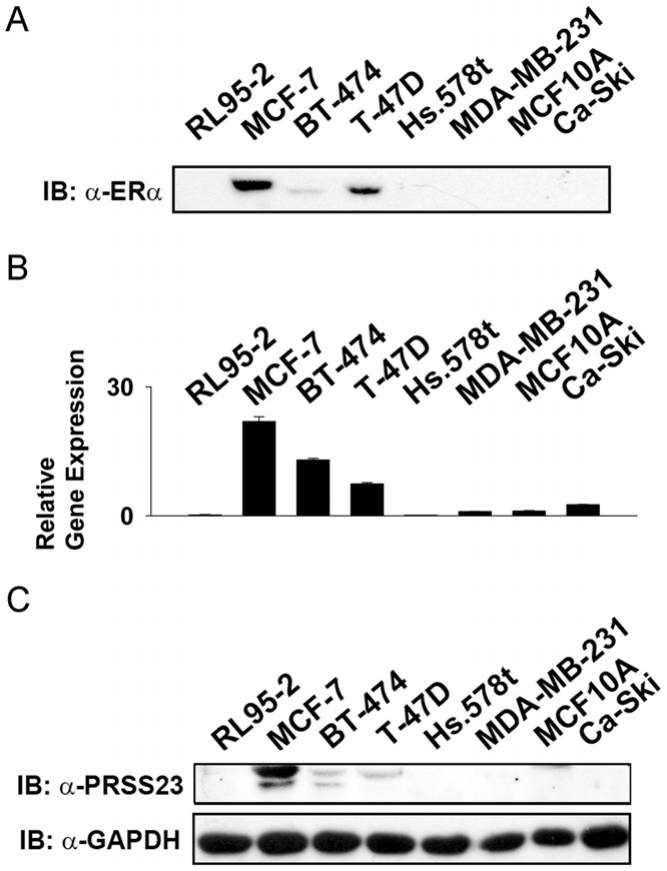
Expression analyses of PRSS23 in human cell lines. Expression levels of PRSS23 as well as ERα were analyzed in eight different cell lines: MCF-7, BT-474, Hs.578t, MDA-MB-231, T-47D (all breast cancer), MCF-10A (mammary epithelial), RL95-2 (endometrial cancer), and Ca-SKi (cervical cancer) cell lines. **A.** Immunoblot analysis showed protein expression level of ERα in these human cell lines. **B.** qRT-PCR analysis showed relative gene expression of PRSS23 mRNA level. **C.** Immunoblot analysis showed protein expression level of PRSS23 and GAPDH in these human cell lines. The cell lysate was loaded 20 µg protein for each well in immunoblot anaylsis. qRT-PCR was performed in duplicate.

Positive expression of endogenous ERα was identified in MCF-7, BT-474, and T-47D cells, which are all ERα-positive cancer cell lines validated by anti-ERα staining. The results showed that ERα was detected in MCF-7, BT-474, and T-47D cells (Fig. 3A). Furthermore, by RT-qPCR survey, the normalized relative gene expression level of PRSS23 was 0.2 in RL95-2 cells, 21.9 in MCF-7 cells, 13.3 in BT-474 cells, 7.44 in T-47D cells, 0.14 in Hs.578t cells, 1 in MDA-MB-231 cells, 1.12 in MCF-10A cells, and 2.58 in Ca-Ski cells (Fig. 3B); all of the expression level was normalized to the PRSS23 mRNA level in MDA-MB-231 cells.

Based on immunoblotting, expression of endogenous PRSS23 was identified in all cell lines utilized in this assay described above by anti-PRSS23, and endogenous GAPDH staining served as the loading control. The results showed that PRSS23 protein expression was detected in ERα-positive MCF-7 cells, BT-474 cells, and T-47D cells (Fig. 3C). Quantification using densitometry analysis revealed the expression level of PRSS23 to be 1 in MCF-7 cells, 0.18 in BT-474 cells, and 0.11 in T-47D cells (expression was normalized to GAPDH expression in the respective cell line). The results indicated that the expression level of PRSS23 was higher in the other cell lines with ERα expression than those without ERα expression. These data from cell line survey also implicated that ERα might upregulate expression of PRSS23 in agreement with the microarray and immunohistochemical studies.

### E_2_ upregulates PRSS23 expression in ERα-positive MCF-7 breast cancer cell

**Figure 4:**
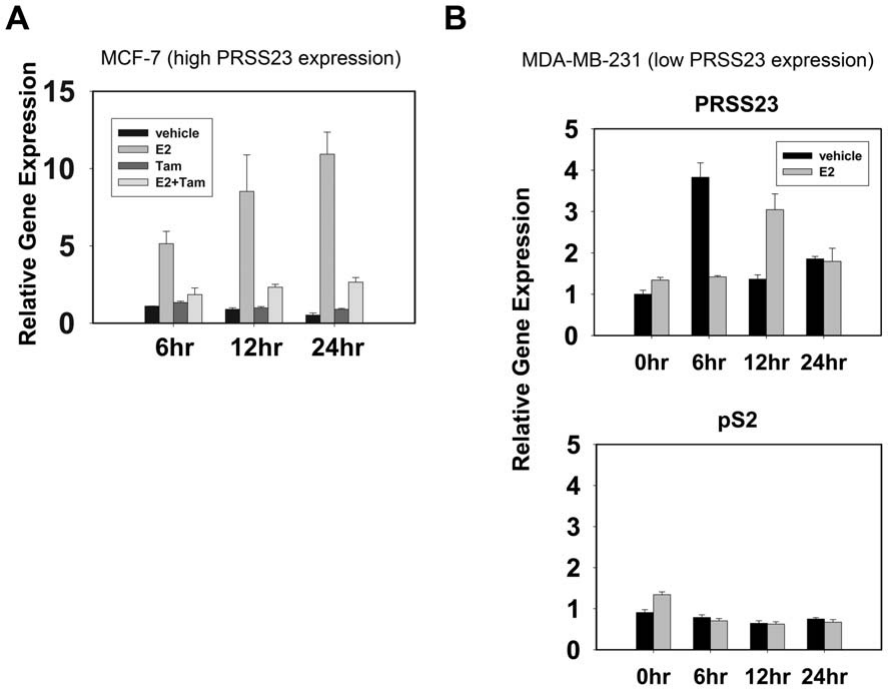
E_2_-activated ERα enhances PRSS23 expression in MCF-7 cells. **A.** MCF-7 cells were treated with 1 nM E_2_, 25 ppm ethanol, 5 µM Tam, and 0.5% dimethyl sulfoxide (DMSO) in phenol-red-free culture medium containing 10% CDS-FBS for 24 h. The bar plots depicted the results of time-lapse profiling of PRSS23 mRNA levels at 6, 12, and 24 h. All experiments were performed in triplicate. The bars represent relative expression levels of PRSS23 after treatment, which was normalized to the level of 6 h-treated cells (mean ± S.E.M.). **B.** MDA-MB-231 cells were treated with 1 nM E_2_ in phenol-red-free culture medium containing 10% CDS-FBS for 24 h. Expression of PRSS23 (upper panel) and pS2 (lower panel) was evaluated by qRT-PCR at 0, 6, 12, and 24 h. The bars represented the gene expression levels of PRSS23 after treatment, which was normalized to the level of untreated cells (mean ± S.E.M.).

After learning that PRSS23 expression was correlated with ERα in breast cancers, we investigated the dynamics of PRSS23 expression induced by estrogen stimulation. We treated the MCF-7 cells with E_2_ and Tamoxifen (Tam) to test whether PRSS23 expression could be enhanced by activated ERα. We found that PRSS23 mRNA expression increased significantly in MCF-7 cells from 6, 12, and 24 h after E_2_ treatment (Fig. 4A). After 24 h of treatment with 1 nM E_2_, PRSS23 mRNA expression was about 10-fold greater than the vehicle control (0.1% DMSO and 25 ppm ethanol). By comparison, PRSS23 mRNA expression was significantly reduced by 5 µM Tam treatment to a similar level as the vehicle controls. In addition, Tam alone did not upregulate PRSS23 mRNA levels in MCF-7 cells compared with the vehicle control.

To confirm whether estrogen is indeed not able to upregulate PRSS23 expression in ERα-negative cancer cells, we treated MDA-MB-231 (ERα-negative) cells with 1 nM E_2_ and measured the mRNA levels of PRSS23 and pS2, with the latter serving as a positive control for estrogen responsiveness [23]. At 0, 6, 12 and 24 h after treatment, no significant correlationship was observed in gene expression levels of PRSS23 in MDA-MB-231 cells treated with 1 nM E_2_ compared with vehicle treated control (Fig. 4B upper panel) as compared to pS2 (Fig. 4B lower panel). Although the *PRSS23* gene expression level in E_2_-treated cells is 3-fold higher than that of vehicle treated control at 12 h. We hypothesized PRSS23 expression might be regulated by alternative signaling pathway in ER-negative MDA-MB-231 cells. Taken together, these data suggest that PRSS23 expression is indeed primarily regulated by estrogen signaling in ER-positive breast cancer cells.

### Overexpression of ERα enhances PRSS23 expression in MCF-7 cells

**Figure 5:**
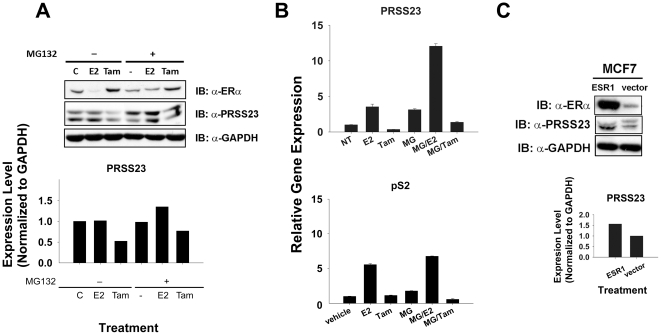
Increased ERα level upregulated PRSS23 expression in MCF-7 cells. **A.** MCF-7 cells were treated with 1 nM E2, 1 µM Tam, or vehicle (0.1% DMSO) in the absence or presence of 5 µg/ml MG-132. The immunoblot showed the protein levels of ERα, PRSS23 and GAPDH (20 µg lysate protein/well). The bar chart represents the normalized protein level of PRSS23 to GAPDH loading control after 12 h treatment with 1 nM E2, 1 µM Tam, or vehicle. **B.** The bars represent gene expression levels of PRSS23 (upper panel) and pS2 (lower panel) in mean ± S.E.M. **C.** MCF-7 cells were transfected with ESR1 or vector for 12 h in culture medium containing 10% FBS. Protein levels of ERα and PRSS23 in the transfected MCF-7 cells was determined by immunoblot analysis (20 µg lysate protein/well). The bar chart represented protein expression levels of PRSS23 normalized to GAPDH. All experiments were performed in duplicate.

Based on the results described above, we hypothesized that ERα protein level is relevant to the expression of PRSS23. Previous studies have shown that ERα upregulates gene expression of *pS2* and *CTSD* by recruiting estrogen, and E_2_-bound ERα is prone to immediate ubiquitin-dependent degradation by the 20S proteasomes after stabilizing transcription initiation [24], [25], [26], [27]. To assay whether a similar ERα stability issue could affect PRSS23 mRNA expression, we used MG-132 to perturb intracellular proteasome activity in MCF-7 cells. When proteasome activity was not disrupted by MG-132, ERα level appeared to be reduced in E_2_-treated MCF-7 cells due to ubiquitin-dependent degradation (Fig. 5A). Treatment with the proteasome inhibitor MG-132, however, blocked the decrease in E_2_-induced ERα protein levels. Furthermore, Tam could not induce ERα degradation in MCF-7 cells, which was consistent with findings from a previous study [24]. Our results (Fig. 5A) indicated that cotreatment with MG-132 and E_2_ for 12 h could significantly increase the PRSS23 protein level in MCF-7 cells (near 1.5-fold) as compared with E_2_ treatment alone in the assay. Moreover, we also found the protein level of PRSS23 significantly decreased near 0.5-fold to 0.6-fold in Tam-treated MCF-7 cells whether MG-132 is present in the medium or not.

We also found that cotreatment with MG-132 and E_2_ for 12 h could increase the PRSS23 mRNA level (3-fold; Fig. 5B upper panel) and pS2 level (1.3-fold; Fig. 5B lower panel) in MCF-7 cells compared with treatment with E_2_ alone. Although MG-132 enhanced the PRSS23 mRNA level by 2.5-fold, cotreatment with MG-132 and Tam reduced PRSS23 mRNA to a level similar to untreated MCF-7 cells. These results suggest that the stability of E_2_-activated ERα upregulates PRSS23 mRNA expression, whereas Tam-inactivated ERα does not stimulate PRSS23 expression.

To clarify whether accumulation of ERα contributes exclusively to the upregulation of PRSS23 expression, we ectopically expressed ERα in MCF-7 cells. Fig. 5C shows that the PRSS23 protein level was increased ∼1.5-fold in MCF-7 cells when ectopic ERα was overexpressed. As expected, the enhancement was not observed in the vector-only controls. Thus, these data suggest the activity and stability of ERα are important for the regulation of PRSS23 expression in MCF-7 cells.

### E_2_ activates ERα to upregulate PRSS23 expression through an upstream promoter region

**Figure 6:**
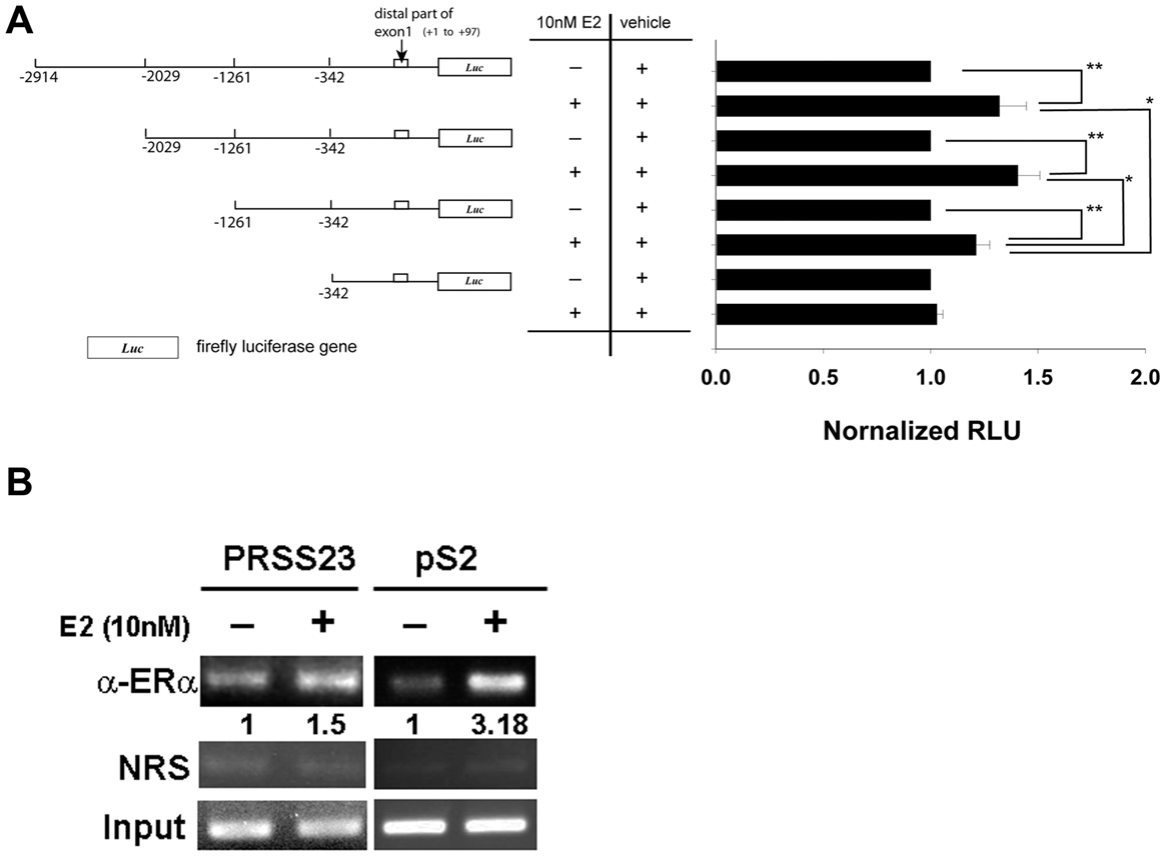
ERα upregulated PRSS23 expression through its upstream promoter region at −2029 to −342 bp in MCF-7 cells. **A.** This scheme depicts the *p*GL3-basic constructs containing the truncated *PRSS23* promoters. Hormone-starved MCF-7 cells were separately transfected with the constructs for 12 h in phenol-red-free medium containing 10% CDS-FBS. Transfected MCF-7 cells were treated with 10 nM E_2_ or vehicle control (250 ppm ethanol) for 16 h in phenol-red-free medium containing 10% CDS-FBS. Level of relative luciferase units (RLUs) were normalized to ethanol control. * *p*<0.05 and ** *p*<0.01 by the Mann-Whitney U test. **B.** Hormone-starved MCF-7 cells were treated with vehicle control (250 ppm ethanol) or 10 nM E_2_ for 60 min. The binding of ERα to the upstream promoter region of the *PRSS23* gene and the promoter of *pS2* gene was examined in a ChIP assay. Input control was 10% of original input cell lysate. NRS stands for nonspecific rabbit serum. These results are representative of three individual experiments.

Previous studies have suggested that ERα enhances downstream gene expression through both genomic and non-genomic pathways [19], [28]. In addition, Moggs et al. postulated that a consensus estrogen responsive element is located in the upstream promoter region −2840 to −2828 bp from the translational start site of the *PRSS23* gene [23] To identify the critical estrogen response region in the promoter region upstream of *PRSS23*, we used the genomic sequence from the NCBI Entrez Gene Database to design a set of PCR primers, which were used to subclone various promoter regions along with the upstream regulatory region. Fig. 6A shows the luciferase reporter constructs that we generated, which contained various regions across the *PRSS23* promoter, including −2914 to 97 bp, −2029 to 97 bp, −1261 to 97 bp, and −391 to 97 bp. We transfected MCF-7 cells with individual reporter construct containing these variable promoter sequences to screen for the most critical estrogen responsive region. Interestingly, the normalized luciferase activities of the −2914 to 97 bp, −2029 to 97 bp, and −1261 to 97 bp constructs increased by 35%, 40%, and 20% in E_2_-treated MCF-7 cells compared with vehicle-treated cells, respectively (*p*<0.01, Fig. 6A). By comparison, the normalized luciferase activity of the construct containing the −342 to 97 bp promoter region did not show significant enhancement in E_2_-treated cells. Interestingly, the difference in the luciferase activities between the −2914 to 97 bp and −2029 to 97 bp constructs was not significant in the presence of E_2_ (*p*>0.05); however, the luciferase activity of the −1261 to 97 bp construct was 11% lower than the activity of the −2914 to 97 bp construct (*p*<0.05). A more profound difference was observed between the −1261 to 97 bp construct and the −2029 to 97 bp construct (*p*<0.05), in which the activity of −2029 to 97 bp construct was increased by 15% compared to that of −1261 to 97 bp construct in the presence of E_2_. Taken together, these results suggest that ERα upregulates *PRSS23* promoter activity through different elements in the region within −2029 to −342 bp instead of through the hypothetical ERE (−2840 to −2828 bp).

Based on the findings with the promoter region constructs, we used ChIP assays to examine whether ERα directly binds to promoter region upstream of the *PRSS23* gene. The *pS2* gene served as a positive control. Fig. 6B shows that binding of ERα to the upstream promoter region was enhanced in 10 nM E_2_-stimulated MCF-7 cells after 60 min of treatment. Compared with vehicle-treated controls, the strength of the interaction of ERα with the upstream promoter region of the *pS2* gene was 3-fold higher, and that of *PRSS23* gene after 60 min of treatment was 1.5-fold higher, which indicates that ERα upregulates PRSS23 expression through direct interaction via its upstream promoter region.

### PRSS23 expression is associated with estrogen-induced proliferation in MCF-7 cells

**Figure 7:**
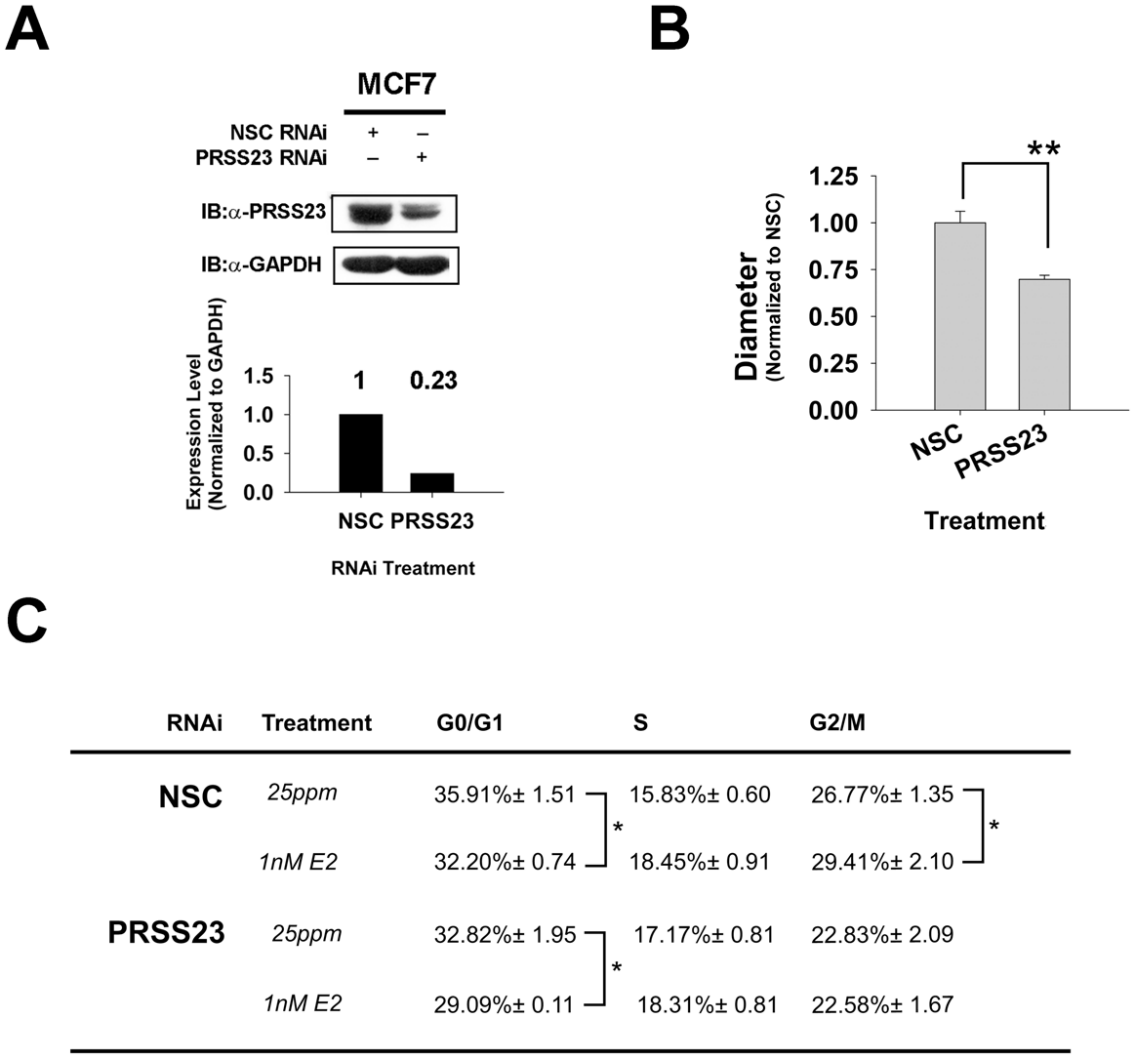
PRSS23 knockdown reduced estrogen-driven MCF-7 cell proliferation. **A.** The PRSS23 knockdown efficacy in MCF7 cells treated with nonspecific control (NSC) or PRSS23-specific RNAi was validated by immunoblotting. GAPDH was used as the loading control. The bar chart shows the normalized protein level of PRSS23 in NSC and PRSS23 RNAi cells. **B.** The tumor sphere formation abilities of cells were evaluated in the soft-agar tumor formation assay in the presence or absence of PRSS23 RNAi. The upper panel shows a representative picture of tumor sphere formation in 0.4% soft-agar (scale bar is 200 µm). The bar chart shows that normalized diameter of examined tumors (n≥50). The results are the average of two individual experiments. **C.** After culturing in phenol-red-free medium containing 0.5% CDS-FBS for 48 h, the cells were stimulated with 20% CDS-FBS and 1 nM E_2_ or 25 ppm ethanol for 24 h. The table shows the DNA distribution profile of the examined cells. Each value is the average count of the cells in three individual experiments. * *p*<0.05 and ** *p*<0.01 by the Mann-Whitney U test.

Our earlier immunohistochemical data revealed that PRSS23 was located in the cell nucleus of breast cancer cells. Thus, we used an RNAi knockdown approach to examine cancer cell function could be affected by PRSS23 on breast cancer cell proliferation. The efficacy of RNAi-mediated PRSS23 knockdown was initially determined by immunoblot analysis (Fig. 7A). We found that PRSS23 protein levels could be reduced by ∼77% in cells treated with RNAi directed against PRSS23 compared with cells treated with the non-silencing control (NSC). After confirming the PRSS23 knockdown, we used the PRSS23 knockdown MCF-7 cells in colony formation assays. The cells were cultured in 0.4% soft agar with 10% fetal bovine serum (FBS) without hormone deprivation for 6 days (Fig. 7B, upper panel), and the size of each tumor particle was evaluated by diameter. When sufficient E_2_ was present, the average diameter of tumors formed in PRSS23 knockdown cells was 30% less than the average diameter in NSC cells-forming tumors (*p*<0.01; Fig. 7B, bar graph).

We also performed flow cytometry analysis to map the DNA distribution profile of MCF-7 cells for cell cycle analysis. We initially examined NSC control cells after 24 h stimulation with 20% FBS, either in the absence or presence of E_2_. Compared with the ethanol vehicle-control cells, treatment with 1 nM E_2_ decreased cell counts at the G0/G1 phase from 35.91% to 32.20%, which represented a 10% reduction (Fig. 7C). In addition, the S and G2/M phases each showed a 16.5% (15.83%→18.45%) and a 9.7% (26.77%→29.41%) increase, respectively, in the E_2_-treated cells compared with the control cells.

After knockdown of PRSS23, the 1 nM E_2_ treatment still caused an 11.4% decrease (32.82%→29.09%) in the G0/G1 phase; however, there was only showed a 6.6% increase (17.17%→18.31%) in the S phase and a negligible 0.25% reduction (22.83%→22.58%) in the G2/M phase. Thus, these results indicate that PRSS23 is associated with E_2_–induced MCF-7 cell proliferation.

## Discussion

The present study investigated which proteases were associated with ERα in breast cancer. Bioinformatic analyses on breast cancer microarray datasets published by van't Veer et al. [21] revealed that PRSS23 is one of the most highly expressed proteases linked to ERα expression. Histopathological assays and surveys of cancer cell lines further confirmed PRSS23 expression was significantly increased in ERα-positive breast cancers, and PRSS23 expression was upregulated by ERα-mediated transcriptional regulation. We also investigated the functional role of PRSS23 and found that PRSS23 may regulate DNA replication during cancer cell proliferation, which highlights PRSS23's potential as a novel target for breast cancer therapy.

Proteases are known to play diverse roles in physiology and pathology. Thus, it would not be surprising if some proteases participated in estrogen-dependent breast tumor cell growth, differentiation, and progression. For instance, cathepsin D (CTSD), which is an estrogen-inducible lysosomal protease identified in breast cancer, is considered to be a critical factor in mediating apoptosis of cancer cells, neurodegeneration, and development regression. Accumulating studies have provided evidence that protein levels of CTSD are an independent biomarker for better prognostic outcome in various cancers [28], [29], [30], [31], [32], [33]. In addition, the results reported in the present study suggest that PRSS23 expression is upregulated by estrogen-activated ERα in MCF-7 cells. Therefore, it is plausible to hypothesize that protein levels of PRSS23 might also serve as an independent prognostic factor for breast cancer. Due to case number limited case numbers, we were not able to resolve the underlying difference in PRSS23 and ERα across the various subtype that could help to subtype breast cancers with distinct prognostic outcomes; however, we were able to validate the association between ERα status and high PRSS23 expression with statistical confidence. Thus, when a sufficient number of breast cancer cases are available, further investigation should be undertaken to explore the importance of PRSS23 in breast cancer patients with different ERα status and adjuvant chemotherapy.

Estrogen can stimulate the transactivity of ERα to upregulate downstream gene expression either through direct binding to the ERE in target genes or through coregulation with other transcription factors [34], [35]. Thus, it is interesting to determine which route is involved in regulation of PRSS23 expression. Our results from luciferase reporter assays indicate that E_2_ stimulates PRSS23 expression in MCF-7 cells through the upstream promoter region **−**2029 to −342 bp. In addition, the ChIP assays showed that E_2_ upregulates *PRSS23* promoter activity by activating ERα. Interestingly, previous studies have revealed that DNA binding domain of ERα is dispensable for ERα-mediated upregulation of *PRSS23* gene expression in MCF-7 cells while E_2_ is present [4]. According to our finding in the promoter activity assay and ChIP assay, the promoter activity of *PRSS23* gene induced by E_2_ treatment is statistically significant (*p*<0.05) but not particularly striking like that of canonical estrogen-induced genes, including *pS2* and *CTSD*. However, our results implied that PRSS23 expression is upregulated by ERα through not only the genomic pathway but also other non-genomic pathway, which shall be investigated in future studies. At least, these results suggest that ERα may upregulate PRSS23 expression by interacting with other transcription factors at −2029 to −342 bp in the promoter region instead of the hypothetical ERE [23] in genomic pathway.

The anti-PRSS23 staining pattern in the immunohistochemical studies of the patient specimens revealed that PRSS23 is found in the cell nuclei of breast cancer cells and in normal stromal and endothelial cells of peripheral tissues. The nuclear localization of PRSS23 has been confirmed by subcellular fractionation studies (unpublished data). Interestingly, another group used yeast two-hybrid screening to show that PRSS23 might interact with NCAPD3 (non-SMC Condensin II complex subunit D3), which has been shown to play a significant role in mediating chromosome condensation, segregation, and DNA repair during S phase to prophase of the cell cycle [36], [37], [38]. Based on these findings, we hypothesized that PRSS23 might be involved in estrogen-driven mechanisms to mediate chromosome replication of ERα-positive breast cancer cells. Although further investigation is needed to resolve the detailed molecular mechanisms and interactions involved, we propose that PRSS23 participates in the regulation of breast cancer proliferation.

In conclusion, the present study demonstrated the close relationship between PRSS23 and estrogen/ERα signaling in breast cancer, which might serve as the basis for developing PRSS23 into a novel prognostic or therapeutic target for breast cancer.

## Materials and Methods

### Ethics statement

All human specimens were encoded to protect patient confidentiality and processed under protocols approved by the Institutional Review Board of Human Subjects Research Ethics Committee of Mackay Memorial Hospital, Taipei City, Taiwan and local law regulation. Breast cancer tissues along with their relative normal counterparts were obtained from residual sample bank of Mackay Memorial Hospital and reviewed and provided without linkage to patients' information by pathologists (10MMHIS135). Written consents for placental tissue were obtained from the patient for the present study (MMHIS137).

### Cell culture, cell transfection and RNA interference

MCF-7, MDA-MB-231, Hs.578t and Ca-SKi cells were cultured in RPMI 1640 medium (Cassion Laboratories, North Logan, UT, USA) supplemented with 10% fetal bovine serum (Invitrogen, Carlsbad, CA, USA), 2 g/l sodium bicarbonate, 15 mM HEPES, and 1 mM sodium pyruvate; T-47D were cultured in medium supplemented with 0.2 U/ml insulin (Sigma-Aldrich, St. Louis, MO, USA). BT-474 and RL95-2 cell lines were cultured according to the instructions of American Type Culture Collection. The MCF-10A cell culture has been previously documented [39].

For transfections, plasmids were delivered with jetPRIME transfection reagent (PolyPlus, Yvelines, France) according to the manufacturer's instructions. The RNAi knockdown system was adopted from the *p*GIPZ vector–based lentivirus system (Open Biosystems, Huntsville, AL, USA), and PRSS23 RNAi sequence is 5′-ACCCAGATTTGCTATTGGATTA-3′. The transfection and transduction procedures followed the manufacturer's instructions.

In estrogen treatment experiments, cultured cells were incubated in phenol-red-free RPMI1640 medium (Cassion Laboratories) with 10% dextran-coated charcoal-stripped fetal bovine serum (CDS-FBS) which was prepared with dextran-coated activated charcoal (Sigma-Aldrich) according to the manufacturer's instructions. 17β-estradiol (E_2_) and tamoxifen (Tam) were all purchased from Sigma-Aldrich Corporation.

### RNA isolation, cDNA synthesis and gene expression quantitation

Total RNA was isolated using TRIzol reagent (Invitrogen) according to the manufacturer's instructions. cDNA was synthesized using a SuperScript III reverse transcriptase kit (Invitrogen) following the manufacturer's instructions. Quantitative real-time polymerase chain reaction (qRT-PCR) was carried out with SYBR green PCR master mix (Applied Biosystems, Carlsbad, CA, USA) using an ABI Prism 7500 sequence detector (Applied Biosystems) following the manufacturer's instructions. RPLP0 served as the control for normalization [40]. The sequences of primer pairs are showed in Table S2.

### Cloning and site-directed mutagenesis

The open-reading frame of ESR1 (Addgene plasmid 11351 [41]) was subcloned into the *p*IRES-ZsGreen vector (Clontech, Mountain View, CA, USA). The open-reading frame of PRSS23 was amplified by high-fidelity PCR (primers are listed in Table S1) and cloned into the *p*IRES-ZsGreen1 vector (Clontech).

DNA fragments of the promoter region containing distal part of exon 1 (−2914 to 97 bp and −391 to 97 bp) were separately amplified by high-fidelity PCR of EcoRV-digested, genomic DNA from human placenta tissue (primers are listed in Table S3). DNA sequence analyses verified that the sequences were identical to those published on the Entrez Genome Database, NCBI. DNA sequences containing *PRSS23* promoter ligated into the *p*GL3-basic vector (Promega, Madison, WI, USA). There are two available unique type-II restriction enzyme cutting sites in the DNA fragment of the promoter–NdeI and PstI. The plasmid *p*GL3-basic-*PRSS23* promoter (−2914 to 97 bp) was separately digested by NheI and NdeI, NheI and PstI (New England BioLabs, Ipswich, MA, USA) to generate the other two different constructs of the *PRSS23* promoter (i.e. −2029 to 97 bp, and −1261 to 97 bp, respectively).

### Promoter luciferase reporter assay

For the luciferase reporter assay, 5×10^4^ cells were cotransfected with the *p*CMV-*Luc* vector (Clontech) and *p*GL3-basic *PRSS23* promoter constructs in 24-well plates. After overnight incubation, cells were subcultured in 96-well plate (∼1×10^4^ per well) and treated with E_2_ for 16 hours. Luciferase activity was evaluated using the Dual-Luciferase Reporter Assay kit (Promega) and the VICTOR^3^ multilabel plate reader (PerkinElmer, Waltham, MA, USA).

### Chromatin immunoprecipitation (ChIP) assay

The detailed ChIP procedures have been described by Fujita et al. [42]. Briefly, immunoprecipitation of the DNA-protein complexes was performed with 5 µg per sample of anti-ERα (clone: F-10) (Santa Cruz Biotechnology, Santa Cruz, CA, USA) and non-specific rabbit serum (Pierce Biotechnology, Rockford, IL, USA). The target DNA fragment of each examined sample was separately amplified by Phire Hot-Start DNA polymerase (Finnzymes, Vantaa, Finland) with the primers of the *PRSS23* promoter (−1388 to −1149 bp), 5′-CCCTTAAAATGGTGGAAAATATCAGTTTCC-3′ and 5′-TACATGAGAAAGCCCTGAACACATTATTGT-3′, and *pS2* ChIP PCR primers [43].

### Membrane immunoblot

Immunoblot have been described in previous studies [44]. The primary antibodies used in the present study were anti-ERα (clone: F-10), anti-GAPDH (Santa Cruz Biotechnology) and the anti-human PRSS23 antibody. The intensities of protein bands in photographs were evaluated by ImageJ software.

### Immunohistochemistry

The histological subtype of each tumor was determined after surgery. The malignancy of infiltrating carcinomas was determined according to the Scarff-Bloom-Richardson classification [45]. The staining procedures were according to Li et al. [46], and images were captured by a TE-2000-E microscope equipped with Nikon D50 digital camera (Nikon, Tokyo, Japan). The intensity of PRSS23 expression in sections was scored following the guidelines of the Allred scoring system [22]. Total Allred scores of samples were analyzed with Fisher's exact test to assess differences between the pathological parameters. Classification of HER2 amplification in breast cancer was performed according to Ellis et al. at 2005 [47].

### Soft-agar colony formation assay

We performed soft agar colony formation assays using low melting temperature agarose, as previous described (Sigma-Aldrich) [48]. The images were captured randomly by TE-2000 inverted microscope equipped with Nikon D50 digital camera (Nikon). The size of tumor was all measured in diameter. The mean tumor sizes of different experiments were all normalized to that of the control group.

### Flow cytometry

The examined cells were harvested by 0.05% trypsin-EDTA solution (Invitrogen). After washed with ice-cold 1X PBS thrice, the cells were fixed with ice-cold 75% ethanol at 4°C for 1 h. The cells were stained in a 1X PBS solution containing 6.7 µM propidium iodide, 0.1 µg/ml RNase A (Invitrogen) in at 37°C for 30 min, and then analyzed in FACSCalibur (BD, Bedford, MA, USA).

### Statistics and data analysis

Microarray data of breast cancer patients were manipulated in MySQL software, and clustering and organization of gene expression were processed with Cluster software from the Eisen lab [49]. The self-organized map was produced by TreeView software. The descriptive statistics of the experimental data were analyzed with Student's *t* test, the Mann-Whitney U test, and Fisher's exact test in the R statistical program.

## Supporting Information

Figure S1 **MCF-7 cells was transfected with ectopic PRSS23 and its expressed was detected by anti-PRSS23 with 20 µg lysate protein/well.** PRSS23 displayed an estimated molecular weight around 47 kDa (indicated by black arrow).
(TIF)

Figure S2 **Immunohistochemical characterization of anti-PRSS23 staining.** **A.** High PRSS23 expression: intensity of nuclear staining of breast cancer cells (black arrow) higher than the intensity of stained peripheral stromal cell (white arrow). **B.** Moderate PRSS23 expression: intensity of nuclear staining of breast tumor cells (black arrow) are equal to the intensities of stained peripheral stromal cells (white arrow) and endothelial cells (blue arrow). **C.** Low PRSS23 expression: intensity of nuclear staining of breast cancer cells (black arrow) are higher than the intensity of stained peripheral stromal cells (white arrow). Scale bar is 200 µm.
(TIF)

Materials and Methods S1 **Anti-PRSS23 Production.**
(DOC)

Table S1 **The primer list of the PRSS23 cloning.**
(DOC)

Table S2 **The primer list of qRT-PCR.**
(DOC)

Table S3 **The primer list for promoter cloning of** ***PRSS23*** **gene.**
(DOC)
