# Supplementary figures and images for: Serine Protease PRSS23 Is Upregulated by Estrogen Receptor α and Associated with Proliferation of Breast Cancer Cells
Source: PLoS One. 2012 Jan 23;7(1):e30397. doi: 10.1371/journal.pone.0030397 (PMC3264607; doi:10.1371/journal.pone.0030397)

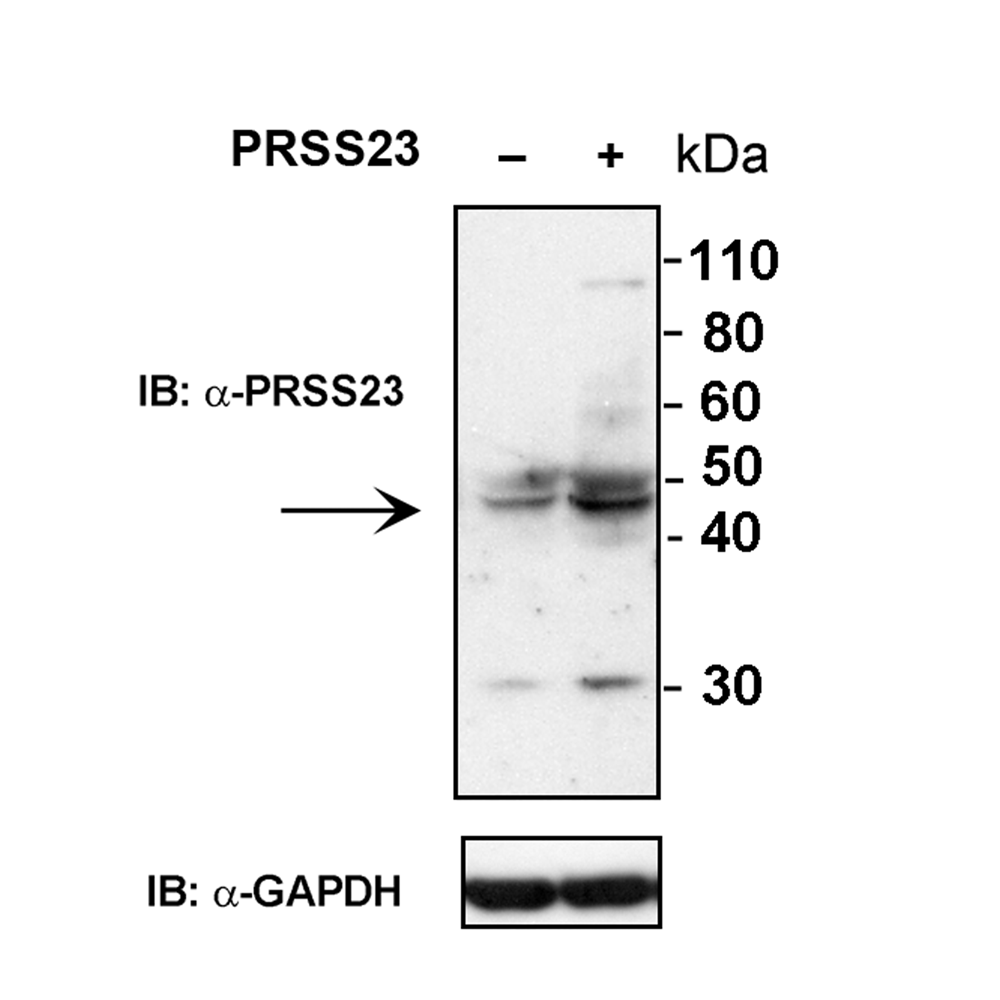

Supplement: Figure S1 — MCF-7 cells was transfected with ectopic PRSS23 and its expressed was detected by anti-PRSS23 with 20 µg lysate protein/well. PRSS23 displayed an estimated molecular weight around 47 kDa (indicated by black arrow). (TIF) [file pone.0030397.s001.tif]

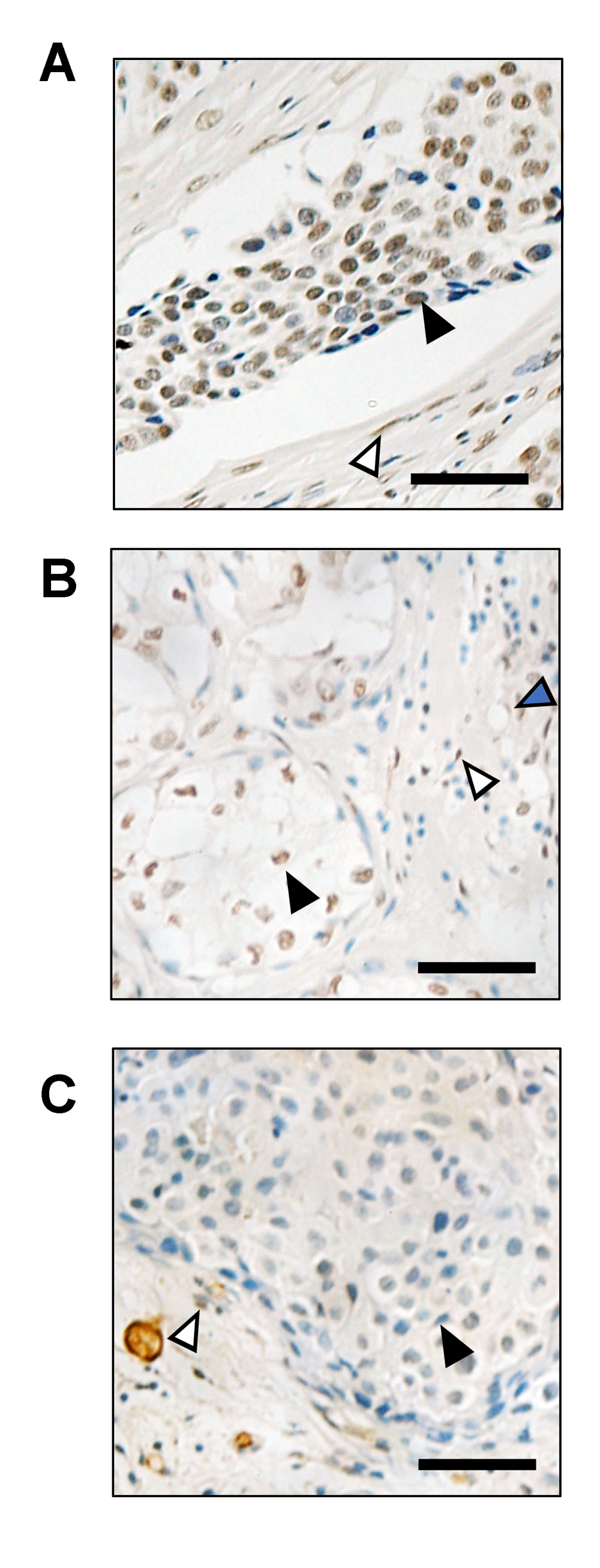

Supplement: Figure S2 — Immunohistochemical characterization of anti-PRSS23 staining. A. High PRSS23 expression: intensity of nuclear staining of breast cancer cells (black arrow) higher than the intensity of stained peripheral stromal cell (white arrow). B. Moderate PRSS23 expression: intensity of nuclear staining of breast tumor cells (black arrow) are equal to the intensities of stained peripheral stromal cells (white arrow) and endothelial cells (blue arrow). C. Low PRSS23 expression: intensity of nuclear staining of breast cancer cells (black arrow) are higher than the intensity of stained peripheral stromal cells (white arrow). Scale bar is 200 µm. (TIF) [file pone.0030397.s002.tif]
