# Supplementary material for: Serine Protease PRSS23 Is Upregulated by Estrogen Receptor α and Associated with Proliferation of Breast Cancer Cells
Source: PLoS One. 2012 Jan 23;7(1):e30397. doi: 10.1371/journal.pone.0030397 (PMC3264607; doi:10.1371/journal.pone.0030397)
Supplement: Materials and Methods S1 — Anti-PRSS23 Production. (DOC) [file pone.0030397.s003.doc]

**SUPPORTING INFORMATION**

**Serine Protease PRSS23 is Upregulated by Estrogen Receptor α and Associated with Proliferation of Breast Cancer Cells**

Hau-Shien Chan, Shing-Jyh Chang, Tao-Yeuan Wang, Hung-Ju Ko,Yu-Chih Lin, Kuan-Ting Lin, Kuo-Ming Chang, Yung-Jen Chuang

**Materials and Methods S1.** **Anti-PRSS23 Production**

DNA fragment of PRSS23 K29-G123 was amplified by high-fidelity PCR (primers are listed in Table S1) and subcloned into the *p*GEX-6p-3 vector (GE Healthcare Life Sciences, Piscataway, NJ). The construct of *p*GEX-6p-3-PRSS23 K29-G123 was used to express (GE Healthcare Life Sciences, Piscataway, NJ) GST-tagged fusion PRSS23 K29-G123 protein in *E. coli* BL21 (DE3) (Invitrogen, Carlsbad, CA). GST-PRSS23 fusion protein was purified with GST HiTrap HF column on ÄKTA prime purification system (GE Healthcare Life Sciences) according the manufacturer’s instructions. The protein served as immunogen to produce anti-PRSS23 rabbit polyclonal antiserum (LTK Laboratories, Taiwan). Anti-human PRSS23 was purified with Protein A HiTrap FF column (GE Healthcare Life Sciences) following the manufacturer’s instructions.
