# Supplementary material for: Serine Protease PRSS23 Is Upregulated by Estrogen Receptor α and Associated with Proliferation of Breast Cancer Cells
Source: PLoS One. 2012 Jan 23;7(1):e30397. doi: 10.1371/journal.pone.0030397 (PMC3264607; doi:10.1371/journal.pone.0030397)
Supplement: Table S1 — The primer list of the PRSS23 cloning. (DOC) [file pone.0030397.s004.doc]

**SUPPORTING INFORMATION**

**Serine Protease PRSS23 is Upregulated by Estrogen Receptor α and Associated with Proliferation of Breast Cancer Cells**

Hau-Shien Chan, Shing-Jyh Chang, Tao-Yeuan Wang, Hung-Ju Ko,Yu-Chih Lin, Kuan-Ting Lin, Kuo-Ming Chang, Yung-Jen Chuang

**Table S1. The primer list of the PRSS23 cloning**

| **Primer sequences** | **Amino acid motif** | **Notes** |
| --- | --- | --- |
| 5’-ATGGCAGGGATTCCAGGGCT-3’ | Full length | Cloning of open reading frame |
| 5’-TCACCCCTCCCTACAATCCAGGT |
| 5’-AAACCCACTTGGCCTGCATA-3’  5’-TCCTGAAGACCCTGAGTCTC-3’ | K_29_-G_123_ | GST protein expression in *E. coli* |
