# Supplementary material for: Serine Protease PRSS23 Is Upregulated by Estrogen Receptor α and Associated with Proliferation of Breast Cancer Cells
Source: PLoS One. 2012 Jan 23;7(1):e30397. doi: 10.1371/journal.pone.0030397 (PMC3264607; doi:10.1371/journal.pone.0030397)
Supplement: Table S2 — The primer list of qRT-PCR. (DOC) [file pone.0030397.s005.doc]

**SUPPORTING INFORMATION**

**Serine Protease PRSS23 is Upregulated by Estrogen Receptor α and Associated with Proliferation of Breast Cancer Cells**

Hau-Shien Chan, Shing-Jyh Chang, Tao-Yeuan Wang, Hung-Ju Ko,Yu-Chih Lin, Kuan-Ting Lin, Kuo-Ming Chang, Yung-Jen Chuang

**Table S2. The primer list of qRT-PCR**

| **Unigene ID** | **Primer sequence** | **Amplicon** | **GenBank**  **accession no.** |
| --- | --- | --- | --- |
| PRSS23 | 5’-ACATCAGTGAAGTTATCCACGGGCT-3’  5’-CCTCGACCACCATCTTTAAACTTGG-3’ | 161 bps  (598 to 758 bp) | NM_007173 |
| RPLP0 | 5'- CGGATTACACCTTCCCACTT-3'  5'- CCGACTCTTCCTTGGCTTC-3' | 150 bps  (994 to 1087 bp) | NP_444505 |
