# Supplementary material for: Serine Protease PRSS23 Is Upregulated by Estrogen Receptor α and Associated with Proliferation of Breast Cancer Cells
Source: PLoS One. 2012 Jan 23;7(1):e30397. doi: 10.1371/journal.pone.0030397 (PMC3264607; doi:10.1371/journal.pone.0030397)
Supplement: Table S3 — The primer list for promoter cloning of PRSS23 gene. (DOC) [file pone.0030397.s006.doc]

**SUPPORTING INFORMATION**

**Serine Protease PRSS23 is Upregulated by Estrogen Receptor α and Associated with Proliferation of Breast Cancer Cells**

Hau-Shien Chan, Shing-Jyh Chang, Tao-Yeuan Wang, Hung-Ju Ko,Yu-Chih Lin, Kuan-Ting Lin, Kuo-Ming Chang, Yung-Jen Chuang

**Table S3. The primer list for promoter cloning of *PRSS23* gene**

| **Amplified region** | **Primer sequence** | **Amplicon** |
| --- | --- | --- |
| -2914 to 97 bp | 5'-GCTAGCGAGACCAGATGAAGGACTGTT-3' '  5'-TCTAGACCATGGCAGGGCCACCAATGCTTT-3' | 3012 bps |
| -391 to 97 bp | 5'- GCTAGCAAAGCATTGGTGGCCCTGCCATGG -3'  5'-TCTAGACCATGGCAGGGCCACCAATGCTTT-3' | 494 bps |
